# Anti-Hyperglycemic Agents and New-Onset Acute Myocardial Infarction in Diabetic Patients with End-Stage Renal Disease Undergoing Dialysis

**DOI:** 10.1371/journal.pone.0160436

**Published:** 2016-08-11

**Authors:** Ting-Tse Lin, Chih-Chen Wu, Yao-Hsu Yang, Lian-Yu Lin, Jiunn-Lee Lin, Pau-Chung Chen, Juey-Jen Hwang

**Affiliations:** 1 Department of Internal Medicine, National Taiwan University Hospital Hsin-Chu Branch, Hsin-Chu, Taiwan; 2 Institute of Biomedical Engineering, National Chiao-Tung University, Hsinchu, Taiwan; 3 Department for Traditional Chinese Medicine, Chang Gung Memorial Hospital Chia-Yi, Taiwan; 4 Institute of Occupational Medicine and Industrial Hygiene, National Taiwan University College of Public Health, Taipei, Taiwan; 5 Center of Excellence for Chang Gung Research Datalink, Chang Gung Memorial Hospital, Chiayi, Taiwan; 6 School of Traditional Chinese Medicine, College of Medicine, Chang Gung University, Taoyuan, Taiwan; 7 Division of Cardiology, Department of Internal Medicine, National Taiwan University College of Medicine and Hospital, Taipei, Taiwan; Baker IDI Heart and Diabetes Institute, AUSTRALIA

## Abstract

**Background:**

Diabetes and chronic kidney disease (CKD) are a high-stakes combination for cardiovascular disease. Patients with decreased kidney function and end-stage renal disease (ESRD) have increased risk of hypoglycemia when attaining better glycemic control, leading to higher risk of myocardial infarction (MI). For these patients, which kinds of anti-hyperglycemic agents would be associated with higher risk of MI is not clear.

**Methods:**

We identified patients from a nation-wide database called Registry for Catastrophic Illness, which encompassed almost 100% of the patients receiving dialysis therapy in Taiwan from 1995 to 2008. Patients with diabetes and ESRD were selected as the study cohort. Propensity score adjustment and Cox's proportional hazards regression model were used to estimate the hazard ratios (HRs) for new-onset MI.

**Results:**

Among 15,161 patients, 39% received insulin, 40% received sulfonylureas, 18% received meglitinides and 3% received thiazolidinedione (TZD). After a median follow-up of 1,357 days, the incidence of MI was significant increase in patients taking sulfonylureas (HR = 1.523, 95% confidence interval [CI] = 1.331–1.744), meglitinides (HR = 1.251, 95% CI = 1.048–1.494) and TZD (HR = 1.515, 95% CI = 1.071–2.145) by using patients receiving insulin therapy as the reference group. The risk of MI remains higher in other three groups in subgroup analyses.

**Conclusions:**

In conclusion, among diabetic patients with ESRD undergoing dialysis, the use of sulfonylureas, meglitinides and TZD are associated with higher risk of new-onset MI as compared with insulin.

## Introduction

Diabetes mellitus (DM) is well known to be the leading cause of chronic kidney disease (CKD).[1] Meanwhile, DM is a cardiovascular disease (CVD) equivalent since people with DM carry a two-fold risk of mortality majorly caused by cardiovascular disease (CVD).[2] Also, studies have demonstrated that CKD imparts an extremely high risk of CVD.[3] Therefore, the comorbidity with DM and CKD is a high-stakes combination for cardiovascular complications and mortality.

Initial intensive glycemic control in individuals with newly diagnosed DM has a long-term benefit in decreasing the risk of myocardial infarction (MI).[4] Lowering HbA1c levels to approximately 7.0% reduces the development of macroalbuminuria, and the deterioration of kidney function.[5–7] However, iatrogenic hypoglycemia is a well-recognized consequence of intensive glucose management and excess mortality has been observed with intensive glucose control in the Action to Control Cardiovascular Risk in Diabetes (ACCORD) study.[8] Severe hypoglycemia in the Veterans Affairs Diabetes Trial (VADT) study was proved to be a powerful predictor of cardiovascular death.[9] Action in Diabetes and Vascular Disease: Preterax and Diamicron Modified Release Controlled Evaluation (ADVANCE) trial also showed an association between severe hypoglycemia and adverse study outcomes.[10]

Patients with decreased kidney function have increased risks for hypoglycemia for 2 reasons: (1) decreased clearance of insulin and some of the oral agents used to treat diabetes[11, 12], and (2) impaired kidney gluconeogenesis.[12] It is not known whether different DM treatment agents would bring different outcomes in patients with end stage renal disease (ESRD). The aim of this study is to investigate whether different oral anti-diabetic agents (OADs) or insulin treatment would have impact on the incidence of new-onset MI in patients with ESRD undergoing dialysis therapy.

## Materials and Methods

### Registry data source

This nationwide, retrospective cohort study used integrated medical and pharmacy claims data from National Health Insurance Research Database (NHIRD) in Taiwan. To comply with data privacy regulations, personal identities were encrypted and all data were analyzed in a de-identified manner. All the data used in our study were released and approved by the Collaboration Center of Health Information Application, Ministry of Health and Welfare, Executive Yuan, Taiwan. In Taiwan, ESRD patients undergoing hemodialysis and peritoneal dialysis are categorized as “severe illness”, which were established as a registry system by the NHIRD. The history of diagnosis and procedures was provided as the International Classification of Diseases Ninth Revision Clinical Modification (ICD-9-CM) codes. In addition, this registry system contains nearly complete the medications, procedures, every OPD visits and hospital admission covered by the insurance were recorded in the database. The Bureau of National Health Insurance performs routine validations of the diagnoses by reviewing the original medical charts of all of the patients who applied for catastrophic illness registration.

### Study population

**Fig 1:**
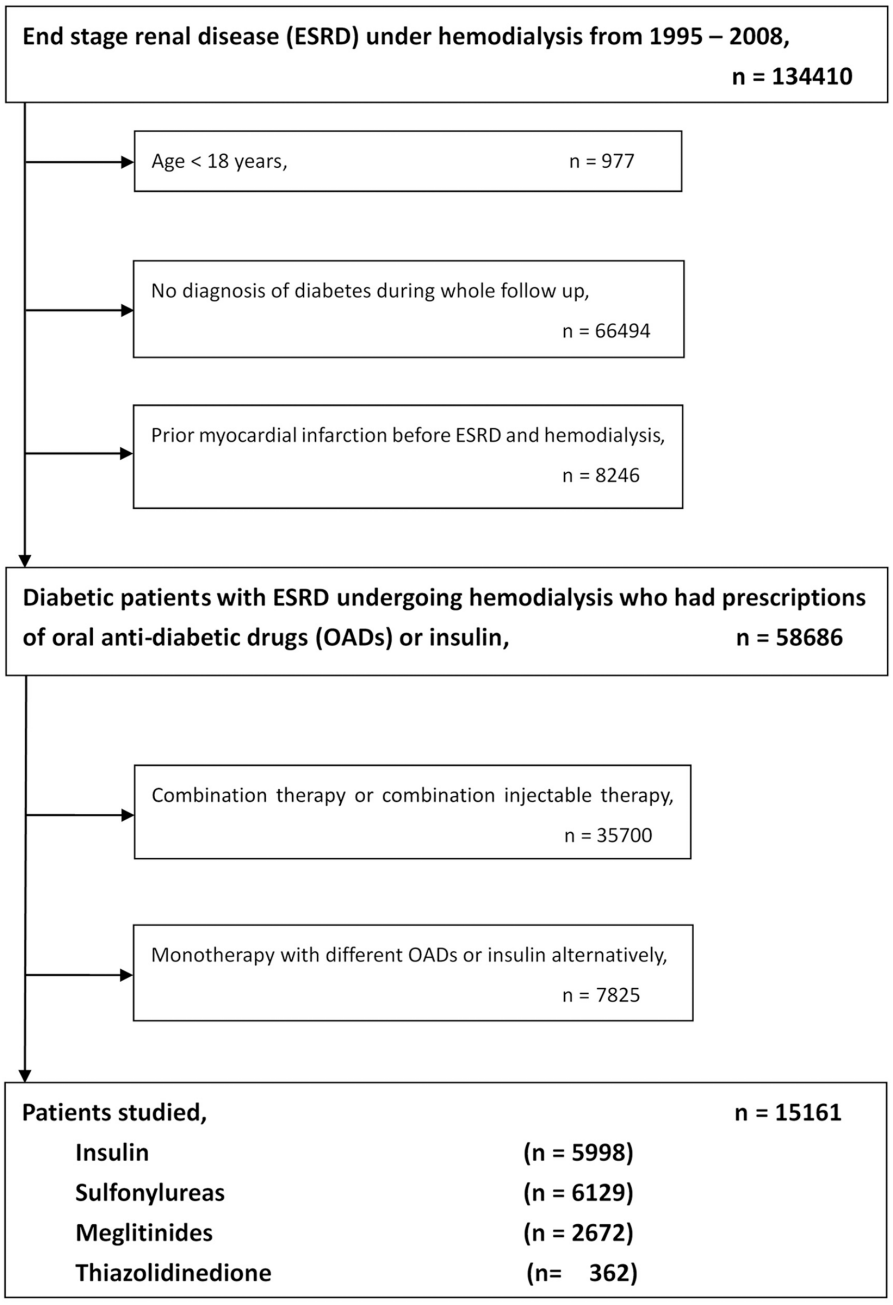
Patient flow diagram.

For the current study, the NHIRD claims database was investigated for the period covering 1995 to 2008. By reviewing ambulatory and inpatient claims data, we included subjects over 18 years of age with diagnosis of diabetes in 1995 and 1996. Like our previous study[13], the date of the first-time that had both a diagnosis of ESRD (ICD9-CM: 585.6, 585.9, 586) and a procedure of hemodialysis (ICD9-CM: 39.93) was operationally defined as the index date. The main exposure of interest was anti-hyperglycemic therapy, identified from prescription claims. The criteria to define the insulin or OADs users were taking these medications for more than 30 days during the following period. Subjects using combination therapy of insulin, sulfonylureas, meglitinides or thiazolidinedione (TZD) and monotherapy but with different OADs or insulin alternatively were excluded. We also excluded first generation sulfonylureas due to small population. We had 5 kinds of second generation sulfonyureas (around 40 of generic drugs with different doses), 3 kinds of meglitinides (around 10 of generic drugs with different doses), 2 kinds of TZD and 5 kinds of insulin. Patients were classified into four groups including patients receiving monotherapy (1) with insulin treatment; (2) with sulfonylureas treatment; (3) with meglitinides treatment and (4) with TZD treatment. A flowchart for the identification of study subjects is shown in Fig 1.

### Covariates and outcomes

Comorbidity was defined by diagnoses at hospital discharge or in clinic records. For the current study, we searched the database to identify the presence of hypertension (HTN) (ICD-9-CM codes: 401.X-405.X), dyslipidemia (272.X), ischemic stroke (ICD9-CM code, 434.X), hemorrhagic stroke (ICD9-CM code, 430.X), coronary artery disease (CAD, ICD9-CM code, 411.X-414.X, V17.3, V81.0), congestive heart failure (CHF) hospitalization (ICD9-CM code, 428.0–428.3, 429.9), peripheral artery disease (PAD) (ICD9-CM code, 250.7, 443.X, 444.2). Medications that were dispensed at time of index date, including angiotensin converting enzyme inhibitors (ACEI), angiotensin receptor blockers (ARB), beta-blockers, calcium channel blockers (CCB), diuretics and statin were identified.

The main purpose of the study was to compare the incidence of newly-developed MI among four groups of patients. Diagnosis of MI was based on ICD-9-CM coding (ICD9-CM code, 410.1–410.9) in any ambulatory visit and discharge diagnoses.

### Statistical analyses

Baseline characteristics, comorbidities, and medication use among four groups were summarized and reported. Person-days of follow-up in each drug use category were computed for all patients in the cohort. One-way ANOVA was used for continuous variables and chi-square test was employed for categorical variables. The crude incidence rates for MI were calculated.

Because of the heterogeneity of four groups, the propensity scores were constructed using multinomial logistic regression to model the receipt of different anti-hyperglycemic agents as a function of baseline patient characteristics.[14] All the background characteristics listed in Table 1, such as age, gender, comorbidities, and medications were included in the multinomial logistic regression model during construction of the propensity scores.

**Table 1:** Demographic and clinical characteristics of study subjects.

	**Insulin**	**Sulfonylureas**	**Meglitinides**	**TZD**
n (%)	5998 (39)	6129 (40)	2672 (18)	362 (3)
Age (mean)	61.5	61.7	62.5*†	62.4*†
18–64 (%)	56.2	58.4*	54.0*†	54.7
65–74 (%)	28.7	28.7	29.5	32.9
≧75 (%)	15.1	12.9*	16.5†	12.4‡
Gender, female %	52.2	48.4*	46.1*†	55.8†‡
Hemodialysis	95.3	97.9*	99.0*†	96.1†‡
HTN, %	96.6	95.1*	97.4†	97.5†
Dyslipidemia	58.4	46.5*	59.0†	56.1†
Ischaemic stroke/TIA, %	21.0	20.0	17.5†	20.7
Haemorrhagic stroke, %	6.5	6.8	4.9†	5.5
CAD, %	48.4	47.8	45.4†	47.0†
PAD, %	40.4	32.6*	33.2*	33.4*
CHF hospitalization, %	33.5	34.2	31.5†	28.5*†
	**Insulin**	**Sulfonylureas**	**Meglitinides**	**TZD**
Medications				
Anti-platelet	30.2	27.2*	29.1	27.9
ACEIs	26.3	30.5*	22.0*†	24.6†
ARBs	35.5	28.5*	37.9*†	39.0†
Beta-blockers	45.5	41.7*	43.9†	45.6
CCBs	71.7	72.7	69.2*†	69.9
Statin	29.0	23.3*	30.3†	39.0*†‡

*p < 0.05 compared with insulin category.

^†^ p < 0.05 compared with sulfonylureas category.

^‡^ p<0.05 compared with meglitinides category

Abbreviations

ACEIs, angiotensin converting enzyme inhibitors; ARB, angiotensin receptor blockers; CAD, coronary artery disease; CCBs, calcium channel blocker; DM, diabetes mellitus; ESRD, end-stage renal disease; HTN, hypertension; HD, hemodialysis; CHF, congestive heart failure; PAD, peripheral artery disease;TIA, transient ischaemic accident; TZD, thiazolidinedione

A Cox proportional hazards regression model was conducted to calculate the hazard ratios (HR) of MI adjusted for baseline demographic data, comorbid conditions and concomitant medication and their 95% confidence intervals using insulin monotherapy as the reference group. The propensity score was applied to reduce the potential bias and to make the four groups more comparable. Using propensity scores as covariates in the regression model could obtain better estimated probability of treatment assignment.[14] To test the consistency of the results, we also did subgroup analyses for different gender, age, presence of HTN, CVD and CHF with adjustment for all confounders. The event-free survival curves of the four groups were illustrated by using the Kaplan-Meier method. The log-rank analysis was applied to test the differences in survival among groups.

All of the analyses were conducted using the Statistical Package for the Social Sciences (SPSS) for Windows, Version 19.0 (SPSS, Inc., Chicago, Illinois). A P value < 0.05 was considered statistically significant.

## Results

### Patient characteristics

There were 15161 patients who met the study inclusion criteria; 5998 (39%) use insulin, while 6129 (40%) used sulfonylureas, 2672 (18%) used meglitinides and 362 (3%) used TZD. The median follow-up time was 1357 days. The algorithm was listed in Fig 1.

Clinical and demographic characteristics were listed in Table 1. Patients in insulin and sulfonylureas group were significantly younger than other two groups and there were significantly less female patients in sulfonylureas and meglitinides group as compared with other two groups. The prevalence of receiving hemodialysis therapy was significantly higher in other three groups than in insulin group. The prevalence of risk factors was higher in insulin, meglitinides and TZD group than in sulfonylureas group, including HTN and dyslipidemia. On the other hand, the prevalence of comorbidities including ischaemic stroke/TIA, hemorrhagic stroke and CAD was higher in insulin, sulfonylureas and TZD group than in meglitinides group. The prevalence PAD was higher in insulin group than in sulfonylureas, meglitinides and TZD group. Finally, CHF hospitalization was lower in meglitinides and TZD groups than in insulin and sulfonylureas group. The baseline characteristics revealed heterogeneous among four groups. In terms of medications, patients receiving anti-platelet drugs were more common in insulin group than other three groups. Patients in sulfonylureas group receiviedmore ACEIs but less ARB and beta-blockers than other three groups. Around 70% patients received CCBs but less common in meglitinides group. At last, more patients received statins in TZD group.

### Main outcome: MI

The median follow-up durations were 1330, 1821, 1261 and 1440 days in insulin, sulfonylureas, meglitinides and TZD groups, respectively. The absolute incidence of MI during the entire follow-up period was more in sulfonylureas (10.4%), meglitinides (6.9%) and TZD (9.6%) groups as compared with that in insulin group (6.3%) (Table 2). After transforming the incidence into patient-years, the incidence was still much lower in insulin group (15.0 per 1000 patient-years) as compared with that in sulfonylureas (20.7 per 1000 patient-years), meglitinides (17.8 per 100 patient-years) and TZD groups (21.5 per 1000 patient-years).

**Table 2:** Incidence of acute coronary syndrome by prescriptions.

	Incidence of acute coronary syndrome				
	Total	Insulin	Sulfonylureas	Meglitinides	TZD
Number of patients	15161	5998	6129	2672	362
Duration of follow-up Median (IQR), days	1357 (807,2274)	1330 (805,2137)	1821 (762,2808)	1261 (855,1871)	1440 (806,2375)
Mean (SD), days	1641 (1198)	1539 (1083)	1839 (1388)	1418 (873)	1640 (1116)
Incident cases—n (%)	1240 (8.2)	380 (6.3)	640 (10.4)	185 (6.9)	35 (9.6)
Incidence per 1000 patient-years	18.2	15.0	20.7	17.8	21.5

Abbreviations: IQR, interquartile range; SD, standard deviation; TZD, thiazolidinedione

**Fig 2:**
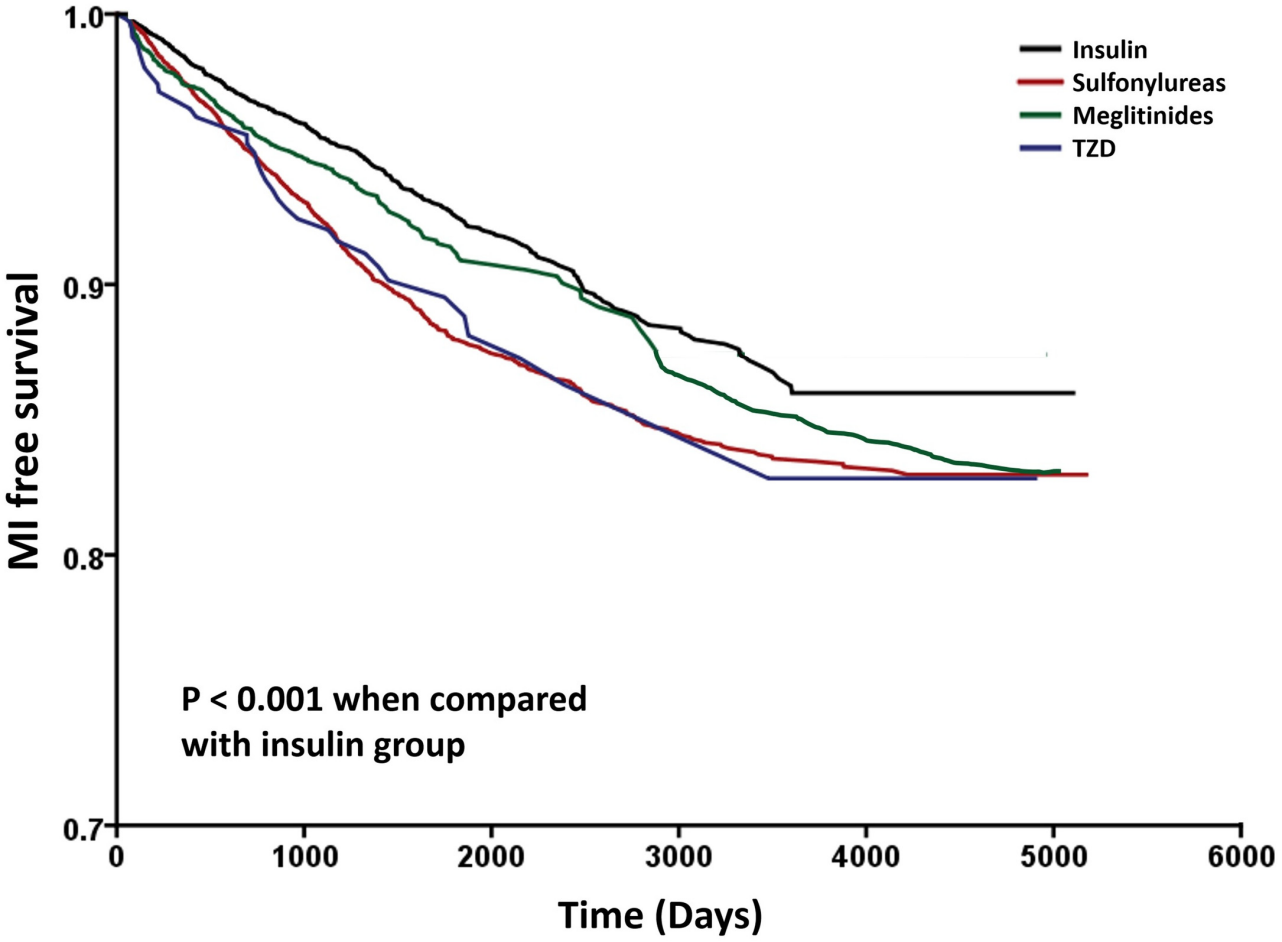
Kaplan–Meier curves showing the development of myocardial infarction (MI) among patients with Insulin (black), sulfonylureas (red), meglitinides (green) and TZD (blue). The log-rank analysis showed significant different (P < 0.001). Abbreviation: TZD, thiazolidinedione.

The results of Cox’s regression analyses were demonstrated in Table 3. After adjusting for potential confounders, in comparison with the insulin group, monotherapy of sulfonylureas (HR: 1.523; 95% CI: 1.331–1.744), meglitinides (HR: 1.251; 95% CI: 1.048–1.494) and TZD (HR: 1.515; 95% CI: 1.071–2.145) were associated with higher risk for developing MI. We observed similar results when we added propensity score in the model (HR: 1.516; 95% CI: 1.325–1.735, HR: 1.245; 95% CI: 1.043–1.486 and HR: 1.509; 95% CI: 1.066–2.137, respectively) (Table 3). The Kaplan-Meier survival curves were illustrated in Fig 2. The log-rank test was significant in sulfonylureas, meglitinides and TZD groups vs. insulin group (P < 0.001).

**Table 3:** Adjusted hazard ratios (95% CI) of developing myocardial infarction in patients receiving sulfonylurea, meglitinides or TZD with insulin treatment as the reference and subgroup analyses.

	**Insulin**	**Sulfonylureas**	**Meglitinides**	**TZD**
**Overall, HR (95% CI)**				
Adjusted HR*	1	1.523 (1.331–1.744)	1.251 (1.048–1.494)	1.515 (1.071–2.145)
Adjusted HR—PS†	1	1.516 (1.325–1.735)	1.245 (1.043–1.486)	1.509 (1.066–2.137)
**Subgroup analyses, HR (95% CI)**				
**Age**				
< 75	1	1.557 (1.359–1.785)	1.213 (1.002–1.469)	1.437 (0.989–2.089)
≧75	1	1.700 (1.119–2.583)	1.560 (1.053–2.555)	2.327 (1.903–5.996)
**Gender**				
M	1	1.661 (1.369–2.016)	1.424 (1.105–1.835)	1.154 (1.012–2.131)
F	1	1.503 (1.261–1.791)	1.084 (0.844–1.392)	1.823 (1.194–2.782)
**HTN**				
Yes	1	1.574 (1.294–1.915)	1.244 (1.044–1.638)	1.907 (1.156–3.145)
No	1	1.573 (1.324–1.870)	1.219 (0.966–1.537)	1.313 (0.810–2.128)
**Hyperlipidemia**				
Yes	1	1.716 (1.455–2.023)	1.294 (1.037–1.614)	1.650 (1.084–2.513)
No	1	1.367 (1.111–1.683)	1.125 (0.837–1.513)	1.410 (0.761–2.613)
	**Insulin**	**Sulfonylureas**	**Meglitinides**	**TZD**
**CAD**				
Yes	1	1.594 (1.380–1.841)	1.251 (1.026–1.526)	1.354 (1.009–2.037)
No	1	1.425 (1.060–1.915)	1.173 (1.094–1.733)	2.257 (1.163–4.381)
**CHF**				
Yes	1	1.491 (1.248–1.783)	1.207 (1.049–1.547)	1.768 (1.089–2.869)
No	1	1.660 (1.375–2.004)	1.247 (0.969–1.605)	1.422 (0.864–2.341)

* Cox proportional regression adjusted for age, gender, HTN, IS, HS, Hyperlipidemia, CHF, PAD, CAD, medication usage (antiplatelet, ACEIs, ARBs, beta-blockers, CCBs and statins)

^†^ Model adjusted for all covariates and propensity score.

Abbreviations

CAD, coronary artery disease; CHF, congestive heart failure; HR, hazard ratio; TZD, thiazolidinedione

**Fig 3:**
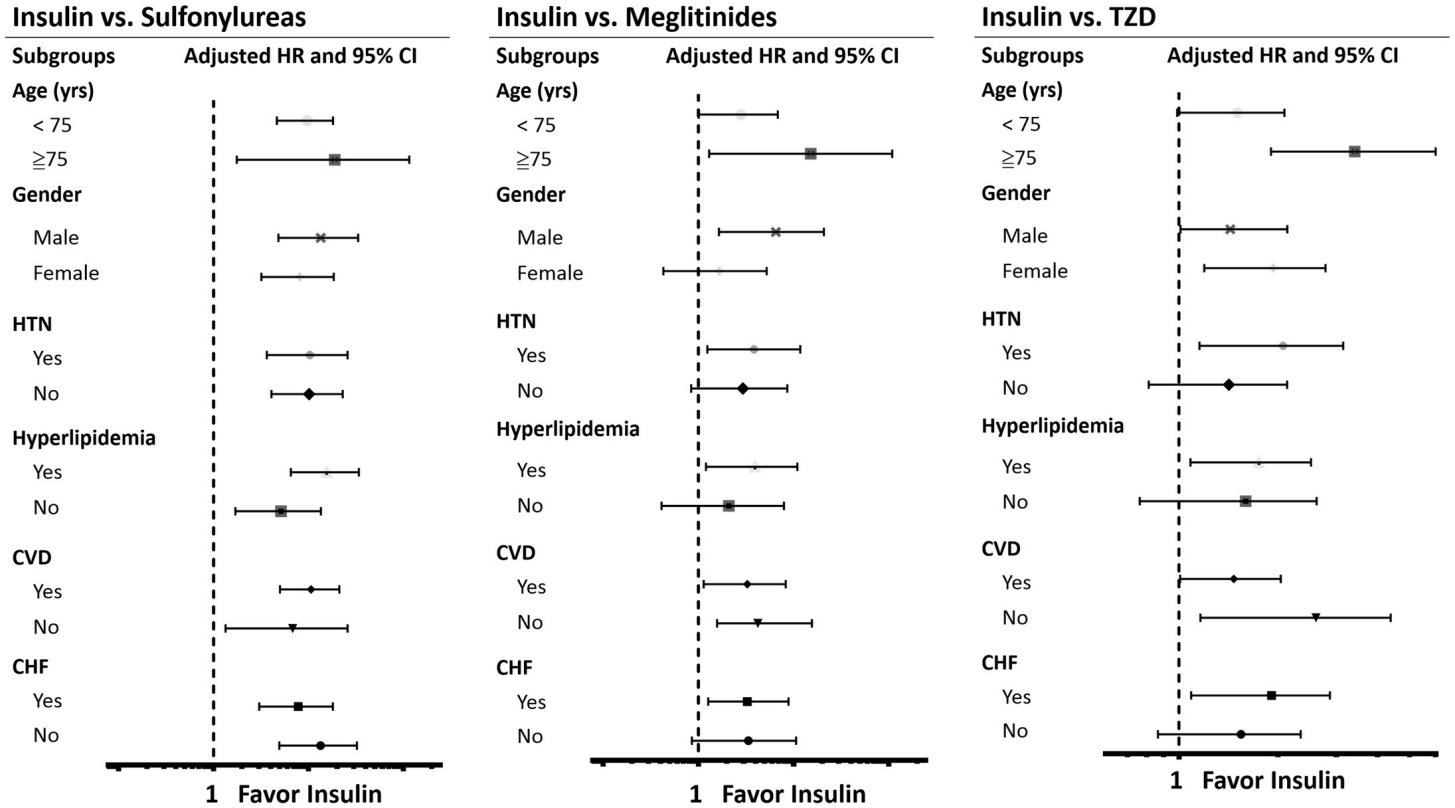
Subgroup analyses. **A**. Hazard ratios of myocardial infarction (MI) in specific subgroups of sulfonylureas treated patients by using insulin as reference group. **B**. Hazard ratios of MI in specific subgroups of meglitinides treated patients by using insulin as reference group. **C**. Hazard ratios of MI in specific subgroups of TZD treated patients by using insulin as reference group. Abbreviations: CI, confidence interval; CVD, cardiovascular disease (combination of coronary artery disease, ischemic stroke, hemorrhagic stroke, peripheral artery disease); CHF, congestive heart failure; HTN, hypertension; HR, hazard ratio; TZD, thiazolidinedione.

The results of subgroup analyses were demonstrated in Table 3 and Fig 3. As shown in Fig 3, compared with insulin usage, sulfonylureas usage was associated with higher risk of MI among the subgroups. For patients treated with meglitinides, there was a similar results except in patients who were female, without HTN, hyperlipidemia and CHF. Likewise, in TZD group, specific subgroups had higher risk of MI, except age below 75 years, patients without HTN, hyperlipidemia and CHF.

## Discussion

This is a nationwide study covering almost 100% of ESRD patients receiving dialysis therapy in Taiwan. Our results showed that patients receiving monotherapy with insulin have a lower risk of MI compared with sulfonylureas, meglitinides and TZD treatment.

Treatment options available for patients with ESRD undergoing dialysis are limited due to safety and tolerability issues, especially for OADs.[15, 16] Insulin injection therapy remains the mainstay of treatment in these patients in order to achieve good glycemic control. [17] Even though recently there were many trials designed to assess the cardiovascular efficacy of current antihyperglycemic drugs, there is no adequate data concerning the use of OADs in dialysis patients, since patients with ESRD were often excluded in these trials.[9, 10, 18] To our knowledge, this study is the first to assess the long-term cardiovascular effect of insulin, sulfonylureas, meglitinides and TZD in patients with ESRD.

Patients with CKD have many concerns when receiving anti-diabetic therapy. For example, several studies have demonstrated that metformin might cause lactic acidosis in patients with advanced CKD.[19, 20] Also, patients treated with first- generation sulfonylureas should be closely monitored to avoid hypoglycemic events because they rely on the kidneys to eliminate both the parent drug and its active metabolites.[21] This is probably the main reason that these sulfonylureas were less prescribed in Taiwan in patients with ESRD and we excluded these patients in this study due to small population. For metglitinides, both repaglinide and nateglinide should be started cautiously with half dosage in advanced CKD patients although an increase in the levels of the active metabolite only happens in nateglinide but not in repaglinide.[22, 23] Rosiglitazone and pioglitazone are cleared by liver and don’t lead to hypoglycemia in patients with CKD.[24] But fluid retention is a major side effect limiting their use in advanced CHF and CKD. For insulin, about one-third insulin is excreted by kidney and patients should closely monitor their glucose levels when treated intensively.[15]

Insulin requirements show a biphasic course in diabetic patients with advanced renal disease. Initially, the glucose control becomes worse as renal function deteriorates, resulting from increasing insulin resistance.[25] As falling of renal function continues, the marked fall in insulin clearance allows a lower dose of insulin to be given, along with an improvement in glucose tolerance.[26, 27] With the starting of hemodialysis, the insulin requirement will depend upon the net balance between the improvement of tissue insulin sensitivity and the restoration of hepatic insulin metabolism. For diabetic patients with ESRD undergoing stable dialysis, it is essential for physicians to carefully adjust the insulin dosage. Unlike insulin, the metabolism of OADs in ESRD patients is far more complicated and the dosage adjustment is difficult.[28] As a result, it is possible that the beneficial effect of insulin on MI in this study is an epiphenomenon of reducing hypoglycemic events.

The annual incidence of new-onset MI is around 2% in this study, which is in accord with the result of a previous Taiwan nation-wide systemic sampling study.[29] In recent three trials assessing the cardiovascular safety and efficacy of dipeptidyl peptidase 4 (DPP-4) inhibitor, the annual incidence of MI is between 1.2 to 1.5% in the cohorts with or without old MI[30, 31] and around 4% in the cohort after acute coronary syndrome.[32] The incidence of MI is somewhat lower in our study since our patients were all receiving dialysis therapy, a condition leading to substantially increased risk of CVD. Since we only included patients receiving monotherapy for DM, it is possible that the lower incidence results from less severity of DM.

ESRD is an extreme manifestation of DM nephropathy, a microvascular complication of the disease mostly results from poor control of blood glucose.[15] In our cohort, a majority of patients (98.7%) had DM prior to dialysis, implying that poor DM control might be the leading cause of ESRD. However around 25% of patients received monotherapy for DM. Previous reports have demonstrated that hemodialysis, mainly by clearing circulating urea, could improve insulin sensitivity and the basal insulin requirements may have a significant 25% reduction of dosage in patient on maintenance dialysis.[33] The increase of insulin sensitivity is probably one of the reasons to explain our observation that a large amount of patients in our study received monotherapy for DM.

In real world, the traditional approach is to initiate monotherapy first, followed by combination therapy to achieve adequate glycemic control. In addition, monotherapy was not enough to maintain decent HbA1c level for these high cardiovascular risk patients. On the other hand, patients who were initially prescribed a drug other than metformin were more likely to need treatment intensification with a second oral glucose-lowering medication or insulin.[34] Nevertheless, monotherapy with insulin may be indicated for initial treatment for some patients, especially those with impaired renal function.[17] Although our patients limited to monotherapy constituted a small portion of DM population, this study could evaluate the pure effect of different anti-hyperglycemic agents on cardiovascular outcomes. The result favoring insulin use may provide the evidence and useful information to physicians to guide selection of initial therapy for ESRD and DM patients.

### Limitations

There are several limitations in this study. First, the current study is a retrospective, nonrandomized study. Second, the imbalance in risk factors among different antihyperglycemic agents’ users in the whole cohort exists and along with treatment selection bias. The result might be still confounded by other underlying disease we did not consider despite we adjusted the confounding factors. Third, the diagnosis of new MI was based on the administrative data reported by physicians, resulting in the concern of accuracy and under-diagnosis. Fourth, we did not analyze the outcome of patients receiving alpha-glucosidase inhibitors and DPP-4 inhibitors, since there were nearly no subjects received monotherapy for alpha-glucosidase inhibitors. As for DPP-4 inhibitors, the licenses were approved in Taiwan after March/2009, which is not in our survey period. Finally, owing to lack of information on glycemic control and data of HbA1c, the beneficial effect of insulin might result from better DM control by insulin rather than avoidance of hypoglycemic events caused by OADs. UK Prospective Diabetes Study (UKPDS) has showed an association between intensive glucose control and a reduced risk of MI over 20 years.[4] However, some clinical trials failed to show beneficial effect of intensive control on cardiovascular outcomes.[35] Though studies of hyperglycemia as a risk factor for cardiovascular events have shown somewhat conflict results, the lack of an analysis of diabetic control weakens the study.

## Conclusions

In this nation-wide cohort study, we showed that in patients with ESRD receiving replacement therapy, insulin usage is associated with less risk of developing MI compared with other OADs.

## References

[1] Zimmet P, Alberti KG, Shaw J. Global and societal implications of the diabetes epidemic. Nature. 2001;414(6865):782–7. 11742409 10.1038/414782a

[2] Haffner SM, Lehto S, Ronnemaa T, Pyorala K, Laakso M. Mortality from coronary heart disease in subjects with type 2 diabetes and in nondiabetic subjects with and without prior myocardial infarction. N Engl J Med. 1998;339(4):229–34. 9673301 10.1056/NEJM199807233390404

[3] Sarnak MJ, Levey AS, Schoolwerth AC, Coresh J, Culleton B, Hamm LL, et al Kidney disease as a risk factor for development of cardiovascular disease: a statement from the American Heart Association Councils on Kidney in Cardiovascular Disease, High Blood Pressure Research, Clinical Cardiology, and Epidemiology and Prevention. Circulation. 2003;108(17):2154–69. 14581387 10.1161/01.CIR.0000095676.90936.80

[4] Holman RR, Paul SK, Bethel MA, Matthews DR, Neil HA. 10-year follow-up of intensive glucose control in type 2 diabetes. N Engl J Med. 2008;359(15):1577–89. Epub 2008 Sep 10. 18784090 10.1056/NEJMoa0806470

[5] Levin SR, Coburn JW, Abraira C, Henderson WG, Colwell JA, Emanuele NV, et al Effect of intensive glycemic control on microalbuminuria in type 2 diabetes. Veterans Affairs Cooperative Study on Glycemic Control and Complications in Type 2 Diabetes Feasibility Trial Investigators. Diabetes Care. 2000;23(10):1478–85. 11023140 10.2337/diacare.23.10.1478

[6] Intensive blood-glucose control with sulphonylureas or insulin compared with conventional treatment and risk of complications in patients with type 2 diabetes (UKPDS 33). UK Prospective Diabetes Study (UKPDS) Group. Lancet. 1998;352(9131):837–53. 9742976

[7] Reichard P, Nilsson BY, Rosenqvist U. The effect of long-term intensified insulin treatment on the development of microvascular complications of diabetes mellitus. N Engl J Med. 1993;329(5):304–9. 8147960 10.1056/NEJM199307293290502

[8] Goff DC Jr., Gerstein HC, Ginsberg HN, Cushman WC, Margolis KL, Byington RP, et al Prevention of cardiovascular disease in persons with type 2 diabetes mellitus: current knowledge and rationale for the Action to Control Cardiovascular Risk in Diabetes (ACCORD) trial. Am J Cardiol. 2007;99(12A):4i–20i. Epub 2007 Apr 12. 17599424 10.1016/j.amjcard.2007.03.002

[9] Duckworth W, Abraira C, Moritz T, Reda D, Emanuele N, Reaven PD, et al Glucose control and vascular complications in veterans with type 2 diabetes. N Engl J Med. 2009;360(2):129–39. 10.1056/NEJMoa0808431. Epub 2008 Dec 17. 19092145

[10] Patel A, MacMahon S, Chalmers J, Neal B, Billot L, Woodward M, et al Intensive blood glucose control and vascular outcomes in patients with type 2 diabetes. N Engl J Med. 2008;358(24):2560–72. 10.1056/NEJMoa0802987. Epub 2008 Jun 6. 18539916

[11] Jonsson A, Rydberg T, Sterner G, Melander A. Pharmacokinetics of glibenclamide and its metabolites in diabetic patients with impaired renal function. Eur J Clin Pharmacol. 1998;53(6):429–35. 9551701 10.1007/s002280050403

[12] Inoue T, Shibahara N, Miyagawa K, Itahana R, Izumi M, Nakanishi T, et al Pharmacokinetics of nateglinide and its metabolites in subjects with type 2 diabetes mellitus and renal failure. Clin Nephrol. 2003;60(2):90–5. 12940610 10.5414/cnp60090

[13] Lin TT, Yang YH, Liao MT, Tsai CT, Hwang JJ, Chiang FT, et al Primary prevention of atrial fibrillation with angiotensin-converting enzyme inhibitors and angiotensin receptor blockers in patients with end-stage renal disease undergoing dialysis. Kidney Int. 2015;88(2):378–85. 10.1038/ki.2015.96. Epub Mar 25. 25807037

[14] Nathan DM, Buse JB, Davidson MB, Ferrannini E, Holman RR, Sherwin R, et al Medical management of hyperglycemia in type 2 diabetes: a consensus algorithm for the initiation and adjustment of therapy: a consensus statement of the American Diabetes Association and the European Association for the Study of Diabetes. Diabetes Care. 2009;32(1):193–203. 10.2337/dc08-9025. Epub 2008 Oct 22. 18945920 PMC2606813

[15] KDOQI Clinical Practice Guidelines and Clinical Practice Recommendations for Diabetes and Chronic Kidney Disease. Am J Kidney Dis. 2007;49(2 Suppl 2):S12–154. 17276798 10.1053/j.ajkd.2006.12.005

[16] Abe M, Okada K, Soma M. Antidiabetic agents in patients with chronic kidney disease and end-stage renal disease on dialysis: metabolism and clinical practice. Curr Drug Metab. 2011;12(1):57–69. 21303332 10.2174/138920011794520053

[17] K/DOQI clinical practice guidelines for cardiovascular disease in dialysis patients. Am J Kidney Dis. 2005;45(4 Suppl 3):S1–153. 15806502

[18] Ismail-Beigi F, Craven T, Banerji MA, Basile J, Calles J, Cohen RM, et al Effect of intensive treatment of hyperglycaemia on microvascular outcomes in type 2 diabetes: an analysis of the ACCORD randomised trial. Lancet. 2010;376(9739):419–30. 10.1016/S0140-6736(10)60576-4. Epub 2010 Jun 30. 20594588 PMC4123233

[19] Salpeter SR, Greyber E, Pasternak GA, Salpeter EE. Risk of fatal and nonfatal lactic acidosis with metformin use in type 2 diabetes mellitus. Cochrane Database Syst Rev. 2010;(4):CD002967 10.1002/14651858.CD002967.pub4. 20091535

[20] Nye HJ, Herrington WG. Metformin: the safest hypoglycaemic agent in chronic kidney disease? Nephron Clin Pract. 2011;118(4):c380–3. 10.1159/000323739. Epub 2011 Feb 16. 21325870

[21] Group. KCW. KDIGO 2012 clinical practice guideline for the evaluation and management of chronic kidney disease. Kidney Int Suppl. 2013;(3):1–150. 10.1038/ki.2013.243 23989362

[22] Nagai T, Imamura M, Iizuka K, Mori M. Hypoglycemia due to nateglinide administration in diabetic patient with chronic renal failure. Diabetes Res Clin Pract. 2003;59(3):191–4. 12590015 10.1016/s0168-8227(02)00242-5

[23] Hasslacher C. Safety and efficacy of repaglinide in type 2 diabetic patients with and without impaired renal function. Diabetes Care. 2003;26(3):886–91. 12610054 10.2337/diacare.26.3.886

[24] Thompson-Culkin K, Zussman B, Miller AK, Freed MI. Pharmacokinetics of rosiglitazone in patients with end-stage renal disease. J Int Med Res. 2002;30(4):391–9. 12235921 10.1177/147323000203000405

[25] Alvestrand A. Carbohydrate and insulin metabolism in renal failure. Kidney Int Suppl. 1997;62:S48–52. 9350680

[26] Weinrauch LA, Healy RW, Leland OS Jr., Goldstein HH, Libertino JA, Takacs FJ, et al Decreased insulin requirement in acute renal failure in diabetic nephropathy. Arch Intern Med. 1978;138(3):399–402. 629634

[27] Adrogue HJ. Glucose homeostasis and the kidney. Kidney Int. 1992;42(5):1266–82. 1453613 10.1038/ki.1992.414

[28] Snyder RW, Berns JS. Use of insulin and oral hypoglycemic medications in patients with diabetes mellitus and advanced kidney disease. Semin Dial. 2004;17(5):365–70. 15461745 10.1111/j.0894-0959.2004.17346.x

[29] Chou MT, Wang JJ, Sun YM, Sheu MJ, Chu CC, Weng SF, et al Epidemiology and mortality among dialysis patients with acute coronary syndrome: Taiwan National Cohort Study. Int J Cardiol. 2013;167(6):2719–23. 10.1016/j.ijcard.2012.06.108. Epub Jul 13. 22795721

[30] Scirica BM, Bhatt DL, Braunwald E, Steg PG, Davidson J, Hirshberg B, et al Saxagliptin and cardiovascular outcomes in patients with type 2 diabetes mellitus. N Engl J Med. 2013;369(14):1317–26. Epub 2013 Sep 2. 23992601 10.1056/NEJMoa1307684

[31] Green JB, Bethel MA, Armstrong PW, Buse JB, Engel SS, Garg J, et al Effect of Sitagliptin on Cardiovascular Outcomes in Type 2 Diabetes. N Engl J Med. 2015;373(3):232–42. 10.1056/NEJMoa1501352. Epub 2015 Jun 8. 26052984

[32] White WB, Cannon CP, Heller SR, Nissen SE, Bergenstal RM, Bakris GL, et al Alogliptin after acute coronary syndrome in patients with type 2 diabetes. N Engl J Med. 2013;369(14):1327–35. Epub 2013 Sep 2. 23992602 10.1056/NEJMoa1305889

[33] Sobngwi E, Enoru S, Ashuntantang G, Azabji-Kenfack M, Dehayem M, Onana A, et al Day-to-day variation of insulin requirements of patients with type 2 diabetes and end-stage renal disease undergoing maintenance hemodialysis. Diabetes Care. 2010;33(7):1409–12. Epub 010 Mar 9. 20215452 10.2337/dc09-2176 PMC2890330

[34] Berkowitz SA, Krumme AA, Avorn J, Brennan T, Matlin OS, Spettell CM, et al Initial choice of oral glucose-lowering medication for diabetes mellitus: a patient-centered comparative effectiveness study. JAMA Intern Med. 2014;174(12):1955–62. 25347323 10.1001/jamainternmed.2014.5294

[35] Skyler JS, Bergenstal R, Bonow RO, Buse J, Deedwania P, Gale EA, et al Intensive glycemic control and the prevention of cardiovascular events: implications of the ACCORD, ADVANCE, and VA diabetes trials: a position statement of the American Diabetes Association and a scientific statement of the American College of Cardiology Foundation and the American Heart Association. Diabetes Care. 2009;32(1):187–92. 10.2337/dc08-9026. Epub 2008 Dec 17. 19092168 PMC2606812

